# Correction: MPRS Duplicated publication

**DOI:** 10.1186/s40902-023-00404-7

**Published:** 2023-10-13

**Authors:** Maxillofacial Plastic and Reconstructive Surgery

**Affiliations:** London, UK

**Correction: Maxillofac Plast Reconstr Surg 41, 35 (2019)**

**https://doi.org/10.1186/s40902-019-0220-6**

**Correction: Maxillofac Plast Reconstr Surg 43, 18 (2021)**

**https://doi.org/10.1186/s40902-021-00305-7**

Following publication of the original article [1, 2], it came to light that due to a publisher error, the same article was published twice in 2019 and 2021. The duplicate articles pertain to the Maxillofacial Plastic and Reconstructive Surgery domain.

The publisher assumes full responsibility and apologizes for these errors.
